# Supplementary material for: Tracing the inner edge of the habitable zone with sulfur chemistry
Source: Sci Adv. 2025 Jan 29;11(5):eadp8105. doi: 10.1126/sciadv.adp8105 (PMC11777254; doi:10.1126/sciadv.adp8105)
Supplement: Supplementary file 1 — Supplementary Discussion References [file sciadv.adp8105_sm.pdf]

Science Advances
AAAS

Supplementary Materials for

Tracing the inner edge of the habitable zone with sulfur chemistry

Sean Jordan *et al.*

Corresponding author: Sean Jordan, jordans@ethz.ch

Sci. Adv. **11**, eadp8105 (2025)
DOI: 10.1126/sciadv.adp8105

This PDF file includes:

Supplementary Discussion
References

Supplementary Discussion

Implications of observing OCS or H_2S

On Venus, SO_2 is observed in the deep atmosphere with a mixing ratio around ~ 150 ppm. The other sulfur gases, OCS and H_2S, have been observed *in situ*, alongside SO_2, at abundances of order ~ 1 – 10 ppm in the deep atmosphere (*64*). These gases are efficiently photochemically destroyed on Venus before they can reach the upper atmosphere where remote sensing could detect their presence. Our results demonstrate that OCS and H_2S are also sensitive to the widely ranging UV fluxes from different M-dwarf host stars. For the low-UV case, OCS and H_2S remain observable in the upper atmosphere, and have spectroscopic features that can be accessed by JWST.

Linking the presence of atmospheric OCS or H_2S to a diagnosis of surface conditions on an exoplanet has not been as well investigated as the presence of atmospheric SO_2 and H_2SO_4 (*19*). SO_2 and H_2SO_4 participate in the oxidised sulfur cycle where sulfur has oxidation state +4 in SO_2 and +6 in H_2SO_4. The sulfur atom in OCS on the other hand has oxidation state 0 and in H_2S has oxidation state -2. The direct connection between the presence of surface water and the reduced sulfur cycle has not yet been well studied and our results suggest that this deserves future investigation. Linking the presence of atmospheric OCS or H_2S to the hydrological cycle of a planet would require investigating the solubility equilibria of OCS and H_2S, and their aqueous chemistry. If the aqueous products of dissolved OCS or H_2S can precipitate out of solution into mineral phases then these sulfur gases may also serve as useful uninhabitability indicators via wet deposition (*19*).

A separate observational implication of the observability of SO_2, OCS and H_2S in the atmospheres of M-dwarf exoplanets is that the relative abundances of each gas could be constrained simultaneously. For low-UV M-dwarf targets, constraining the relative abundance of oxidised and reduced sulfur gases will open a window into a planet's interior oxygen fugacity [e.g., (*41*)], provided that there is sufficient sulfur-outgassing at the surface. If sulfur gases are supplied to the atmosphere via volcanic degassing, then the relative speciation of atmospheric sulfur will depend on the oxygen fugacity of the mantle source from which the melt was derived (*41, 83, 84*). For low-UV M-dwarf exoplanets, the relative proportions of SO_2:OCS:H_2S are effectively undepleted by photochemistry, and so the observed ratio will equal their real ratio in the deep atmosphere. Our result therefore demonstrate that sulfur chemistry can provide a probe of the oxygen fugacity of a

planet's mantle around a low-UV M-dwarf host star, which will be accessible with transmission spectroscopy.

Alternative sources and sinks of sulfur

The prospect of observing spectroscopic features of SO_2, OCS, or H_2S in an exoplanet's atmosphere each have potential caveats, inspired by unknown processes in Venus's sulfur cycle. First, the efficiency of cloud formation in photochemical models of Venus is not sufficient to reproduce the observations of SO_2, H_2O, and H_2SO_4 in the clouds and upper atmosphere [e.g., (*64, 85*)]. In order to reproduce observations, an additional source of hydrogen atoms is required in combination with chemistry beyond only gas phase interactions so that SO_2 can be more efficiently sequestered in the clouds. Previous work has demonstrated how this could be achieved with aqueous chemistry inside the cloud droplets and a source of mineral dust delivered to the clouds (*64*). Alternatively, the observed profile of SO_2 on Venus can be reproduced with the speculative biochemistry of cloud-based microorganisms in the cloud droplets if there is an additional supply of reducing gases in the deep atmosphere (*66, 86*). Upcoming missions to Venus will be able to confirm or deny these hypotheses, however currently these processes cannot be generalised to exoplanets. Due to this limitation, we do not prescribe any additional chemistry beyond the standard gas-phase chemical network. The enhanced depletion of SO_2 that is observed in the clouds of Venus therefore implies that the survival of self-shielding SO_2 above the cloud-layers of exoplanets may be different to the results that we have found. This has the potential to influence the abundances of deep atmosphere SO_2 that are required before above-cloud SO_2 signatures could prevail on targets irradiated by a high-UV M-dwarf that may be accessible with next-generation observatories.

Second is the unknown thermochemical pathway converting some OCS to CO below the cloud base of Venus. Between $\sim 30 - 40$ km altitude, the OCS mixing ratio is observed to decrease from ~ 40 ppm to ~ 1 ppm. Within this altitude range, the CO mixing ratio is observed to increase proportionally, leading to the conclusion that there is unknown OCS chemistry responsible for the conversion to CO and a sulfur-containing product such as a sulfur allotrope. Since the chemical reactions involved remain unknown this cannot be predicted for the case of exoplanets and therefore the observability of OCS in the upper atmosphere is subject to uncertainties on its behaviour in the deep atmosphere. Future work constraining the pressure and temperature dependence of possible

destruction pathways for OCS in the deep atmosphere of Venus will provide valuable insight into the observability of OCS on low-UV M-dwarf exoplanets. The possible destruction pathways of OCS may be possible to verify *in situ* with the upcoming missions exploring Venus's deep atmosphere chemistry (*87*).

Third, there remains the possibility that sulfur-metabolising life could exist in the cloud layer of Venus. Sulfur-metabolising life could catalyse conversions between sulfur-gases based on known sulfur-based energy metabolisms of terrestrial microorganisms (*88, 89*). These metabolic pathways have been thoroughly investigated in the context of Venus's atmosphere previously (*66*) which revealed that SO_2 could be converted to OCS, H_2S, or sulfur allotropes via the biochemical action of life in a H_2SO_4 cloud layer, if such extreme life is possible. On Venus, there has not yet been enough reducing power observed in the deep atmosphere for this conversion to be taking place and thus an abundant biosphere, shaping the atmospheric sulfur chemistry, was ruled out (*66*). However, on sulfur-rich exoplanets this may not necessarily be the case, which therefore poses an alternative mechanism for the scrubbing of SO_2 out of the upper atmosphere and mimicking the presence of surface water oceans. Conversely, if other biosignatures could be detected in combination with this inference then sulfur-metabolising life could be discovered in aerial biospheres in the cloud layers of canonically-uninhabitable exoplanets. This will be a profound avenue of future research if the upcoming Morning Star missions to Venus discover evidence of stable biochemistry in the sulfuric acid cloud droplets (*90*).

Stellar UV variability.

One additional caveat to our results is that M-dwarfs can often exhibit energetic flaring and coronal mass ejection (CME) (*91*). Flares and CMEs are transient events and therefore will influence the steady state background atmospheres which we have restricted ourselves to in this work. If, however, flaring events are sufficiently frequent and energetic then this has the potential to drive different sulfur photochemistry in an exoplanet's atmosphere. Constraining the regions of parameter space where flaring frequency and flaring energy could preclude observations of sulfur chemistry in an exoplanet's atmosphere would be a valuable avenue of future research. We note also that future observations, which could diagnose the photochemical effect of stellar flaring on exoplanet atmospheres, will nonetheless require knowing the chemistry of the background atmosphere that results

under quiescence. Our investigation into the sensitivity of sulfur photochemistry may therefore also be applicable for measuring flare-driven photochemistry around transiently active host stars.

REFERENCES AND NOTES

1. S. R. Kane, R. K. Kopparapu, S. D. Domagal-Goldman, On the frequency of potential venus analogs from *Kepler* data. *Astrophys. J. Lett.* **794**, L5 (2014).

2. J. Yang, N. B. Cowan, D. S. Abbot, Stabilizing cloud feedback dramatically expands the habitable zone of tidally locked planets. *Astrophys. J. Lett.* **771** L45 (2013).

3. M. J. Way, A. D. Del Genio, Venusian habitable climate scenarios: Modeling venus through time and applications to slowly rotating venus-like exoplanets. *J. Geophys. Res. Planets* **125**, e06276 (2020).

4. M. Turbet, T. J. Fauchez, J. Leconte, E. Bolmont, G. Chaverot, F. Forget, E. Millour, F. Selsis, B. Charnay, E. Ducrot, M. Gillon, A. Maurel, G. L. Villanueva, Water condensation zones around main sequence stars. *Astron. Astrophys.* **679**, A126 (2023).

5. I. Baraffe, G. Chabrier, F. Allard, P. H. Hauschildt, Evolutionary models for low-mass stars and brown dwarfs: Uncertainties and limits at very young ages. *Astron. Astrophys.* **382**, 563–572 (2002).

6. R. M. Ramirez, L. Kaltenegger, The Habitable Zones of Pre-main-sequence Stars. *Astrophys. J. Lett.* **797**, L25 (2014).

7. R. Luger, R. Barnes, Extreme water loss and abiotic O2 buildup on planets throughout the habitable zones of M dwarfs. *Astrobiology* **15**, 119–143 (2015).

8. B. S. Konrad, E. Alei, S. P. Quanz, P. Mollière, D. Angerhausen, J. J. Fortney, K. Hakim, S. Jordan, D. Kitzmann, S. Rugheimer, O. Shorttle, R. Wordsworth, LIFE Collaboration, Large Interferometer For Exoplanets (LIFE). IX. Assessing the impact of clouds on atmospheric retrievals at mid-infrared wavelengths with a Venus-twin exoplanet. *Astron. Astrophys.* **673**, A94 (2023).

9. J. K. Barstow, S. Aigrain, P. G. J. Irwin, S. Kendrew, L. N. Fletcher, Telling twins apart: Exo-Earths and Venuses with transit spectroscopy. *Mon. Not. R. Astron. Soc.* **458**, 2657–2666 (2016).

10. G. J. Cooke, D. R. Marsh, C. Walsh, B. Black, J. F. Lamarque, A revised lower estimate of ozone columns during Earth's oxygenated history. *R. Soc. Open Sci.* **9**, 211165 (2022).

11. F. Montmessin, J.-L. Bertaux, F. Lefèvre, E. Marcq, D. Belyaev, J.-C. Gérard, O. Korablev, A. Fedorova, V. Sarago, A. C. Vandaele, A layer of ozone detected in the nightside upper atmosphere of Venus. *Icarus* **216**, 82–85 (2011).

12. E. Marcq, L. Baggio, F. Lefèvre, A. Stolzenbach, F. Montmessin, D. Belyaev, O. Korablev, J.-L. Bertaux, Discovery of cloud top ozone on Venus. *Icarus* **319**, 491–498 (2019).

13. R. Wordsworth, R. Pierrehumbert, Abiotic oxygen-dominated atmospheres on terrestrial habitable zone planets. *Astrophys. J. Lett.* **785**, L20 (2014).

14. J. Krissansen-Totton, J. J. Fortney, Predictions for observable atmospheres of trappist-1 planets from a fully coupled atmosphere-interior evolution model. *Astrophys. J.* **933**, 115 (2022).

15. B. Benneke, S. Seager, Atmospheric retrieval for super-earths: Uniquely constraining the atmospheric composition with transmission spectroscopy. *Astrophys. J.* **753**, 100 (2012).

16. A. H. M. J. Triaud, J. de Wit, F. Klein, M. Turbet, B. V. Rackham, P. Niraula, A. Glidden, O. E. Jagoutz, M. Peč, J. J. Petkowski, S. Seager, F. Selsis, Atmospheric carbon depletion as a tracer of water oceans and biomass on temperate terrestrial exoplanets. *Nat. Astron.* **8**, 17–29 (2024).

17. S. Zieba, L. Kreidberg, E. Ducrot, M. Gillon, C. Morley, L. Schaefer, P. Tamburo, D. D. B. Koll, X. Lyu, L. Acuña, E. Agol, A. R. Iyer, R. Hu, A. P. Lincowski, V. S. Meadows, F. Selsis, E. Bolmont, A. M. Mandell, G. Suissa, No thick carbon dioxide atmosphere on the rocky exoplanet TRAPPIST-1 c. *Nature* **620**, 746–749 (2023).

18. J. Lustig-Yaeger, V. S. Meadows, A. P. Lincowski, A mirage of the cosmic shoreline: Venus-like clouds as a statistical false positive for exoplanet atmospheric erosion. *Astrophys. J. Lett.* **887**, L11 (2019).

19. K. Loftus, R. D. Wordsworth, C. V. Morley, Sulfate aerosol hazes and SO_2 Gas as constraints on rocky exoplanets' surface liquid water. *Astrophys. J.* **887**, 231 (2019).

20. S. Jordan, P. B. Rimmer, O. Shorttle, T. Constantinou, Photochemistry of venus-like planets orbiting K- and M-dwarf stars. *Astrophys. J.* **922**, 44 (2021).

21. M. R. Vidaurri, S. T. Bastelberger, E. T. Wolf, S. Domagal-Goldman, R. Kumar Kopparapu, The outer edge of the venus zone around main-sequence stars. *Planet. Sci. J.* **3**, 137 (2022).

22. R. K. Kopparapu, R. Ramirez, J. F. Kasting, V. Eymet, T. D. Robinson, S. Mahadevan, R. C. Terrien, S. Domagal-Goldman, V. Meadows, R. Deshpande, Habitable zones around main-sequence stars: New estimates. *Astrophys. J.* **765**, 131 (2013).

23. R. K. Kopparapu, E. T. Wolf, G. Arney, N. E. Batalha, J. Haqq-Misra, S. L. Grimm, K. Heng, Habitable moist atmospheres on terrestrial planets near the inner edge of the habitable zone around M dwarfs. *Astrophys. J.* **845**, 5 (2017).

24. J. Leconte, F. Forget, B. Charnay, R. Wordsworth, A. Pottier, Increased insolation threshold for runaway greenhouse processes on Earth-like planets. *Nature* **504**, 268–271 (2013).

25. J. L. Bean, D. S. Abbot, E. M. R. Kempton, A statistical comparative planetology approach to the hunt for habitable exoplanets and life beyond the solar system. *Astrophys. J. Lett.* **841**, L24 (2017).

26. M. Turbet, Two examples of how to use observations of terrestrial planets orbiting in temperate orbits around low mass stars to test key concepts of planetary habitability. arXiv:2005.06512 [astro-ph.EP] (2019).

27. J. Checlair, D. S. Abbot, R. J. Webber, Y. K. Feng, J. L. Bean, E. W. Schwieterman, C. C. Stark, T. D. Robinson, E. Kempton, O. D. N. Alcabes, D. Apai, G. Arney, N. Cowan, S. Domagal-Goldman, C. Dong, D. P. Fleming, Y. Fujii, R. J. Graham, S. D. Guzewich, Y. Hasegawa, B. P. C. Hayworth, S. R. Kane, E. S. Kite, T. D. Komacek, R. K. Kopparapu, M. Mansfield, N. Marounina, B. T. Montet, S. L. Olson, A. Paradise, P. Popovic, B. V. Rackham, R. M. Ramirez, G. Rau, C. Reinhard, J. Renaud, L. Rogers, L. M. Walkowicz, A. Warren, E. T. Wolf, A statistical comparative planetology approach to maximize the scientific return of future exoplanet characterization efforts. *Bull. Am. Astron. Soc.* **51**, 404 (2019).

28. R. J. Graham, R. Pierrehumbert, Thermodynamic and energetic limits on continental silicate weathering strongly impact the climate and habitability of wet, Rocky Worlds. *Astrophys. J.* **896**, 115 (2020).

29. O. R. Lehmer, D. C. Catling, J. Krissansen-Totton, Carbonate-silicate cycle predictions of Earth-like planetary climates and testing the habitable zone concept. *Nat. Commun.* **11**, 6153 (2020).

30. M. Turbet, D. Ehrenreich, C. Lovis, E. Bolmont, T. Fauchez, The runaway greenhouse radius inflation effect. An observational diagnostic to probe water on Earth-sized planets and test the habitable zone concept. *Astron. Astrophys.* **628**, A12 (2019).

31. M. Schlecker, D. Apai, T. Lichtenberg, G. Bergsten, A. Salvador, K. K. Hardegree-Ullman, Bioverse: The habitable zone inner edge discontinuity as an imprint of runaway greenhouse climates on exoplanet demographics. *Planet. Sci. J.* **5**, 3 (2024).

32. J. Lustig-Yaeger, V. S. Meadows, G. Tovar Mendoza, E. W. Schwieterman, Y. Fujii, R. Luger, T. D. Robinson, Detecting ocean glint on exoplanets using multiphase mapping. *Astron. J.* **156**, 301 (2018).

33. K. France, R. O. P. Loyd, A. Youngblood, A. Brown, P. C. Schneider, S. L. Hawley, C. S. Froning, J. L. Linsky, A. Roberge, A. P. Buccino, J. R. A. Davenport, J. M. Fontenla, L. Kaltenegger, A. F. Kowalski, P. J. D. Mauas, Y. Miguel, S. Redfield, S. Rugheimer, F. Tian, M. C. Vieytes, L. M. Walkowicz, K. L. Weisenburger, The MUSCLES treasury survey. I. Motivation and overview. *Astrophys. J.* **820**, 89 (2016).

34. A. Youngblood, K. France, R. O. P. Loyd, J. L. Linsky, S. Redfield, P. C. Schneider, B. E. Wood, A. Brown, C. Froning, Y. Miguel, S. Rugheimer, L. Walkowicz, The MUSCLES treasury survey. II. Intrinsic LYα and extreme ultraviolet spectra of K and M dwarfs with exoplanets. *Astrophys. J.* **824**, 101 (2016).

35. R. O. P. Loyd, K. France, A. Youngblood, C. Schneider, A. Brown, R. Hu, J. Linsky, C. S. Froning, S. Redfield, S. Rugheimer, F. Tian, The MUSCLES treasury survey. III. X-ray to infrared spectra of 11 M and K stars hosting planets. *Astrophys. J.* **824**, 102 (2016).

36. D. J. Wilson, C. S. Froning, G. M. Duvvuri, K. France, A. Youngblood, P. C. Schneider, Z. Berta-Thompson, A. Brown, A. P. Buccino, S. Hawley, J. Irwin, L. Kaltenegger, A. Kowalski, J. Linsky, R. O. P. Loyd, Y. Miguel, J. S. Pineda, S. Redfield, A. Roberge, S. Rugheimer, F. Tian, M. Vieytes, The Mega-MUSCLES spectral energy distribution of TRAPPIST-1. *Astrophys. J.* **911**, 18 (2021).

37. C. Ostberg, S. R. Kane, Z. Li, E. W. Schwieterman, M. L. Hill, K. Bott, P. A. Dalba, T. Fetherolf, J. W. Head, C. T. Unterborn, The demographics of terrestrial planets in the Venus zone. *Astron. J.* **165**, 168 (2023).

38. J. Lustig-Yaeger, G. Fu, E. M. May, K. N. O. Ceballos, S. E. Moran, S. Peacock, K. B. Stevenson, J. Kirk, M. López-Morales, R. J. MacDonald, L. C. Mayorga, D. K. Sing, K. S. Sotzen, J. A. Valenti, J. I. A. Redai, M. K. Alam, N. E. Batalha, K. A. Bennett, J. Gonzalez-Quiles, E. Kruse, J. D. Lothringer, Z. Rustamkulov, H. R. Wakeford, A JWST transmission spectrum of the nearby Earth-sized exoplanet LHS 475 b. *Nat. Astron.* **7**, 1317–1328 (2023).

39. M. Kama, O. Shorttle, A. S. Jermyn, C. P. Folsom, K. Furuya, E. A. Bergin, C. Walsh, L. Keller, Abundant refractory sulfur in protoplanetary disks. *Astrophys. J.* **885**, 114 (2019).

40. X. Byrne, O. Shorttle, S. Jordan, P. B. Rimmer, Atmospheres as a window to rocky exoplanet surfaces. *Mon. Not. R. Astron. Soc.* **527**, 10748–10759 (2023).

41. P. Liggins, S. Jordan, P. B. Rimmer, O. Shorttle, Growth and evolution of secondary volcanic atmospheres: I. Identifying the geological character of hot rocky planets. *J. Geophys. Res. Planets* **127**, e2021JE007123 (2022).

42. R. D. Wordsworth, L. K. Schaefer, R. A. Fischer, Redox evolution via gravitational differentiation on low-mass planets: Implications for abiotic oxygen, water loss, habitability. *Astron. J.* **155**, 195 (2018).

43. S. E. Moran, K. B. Stevenson, D. K. Sing, R. J. MacDonald, J. Kirk, J. Lustig-Yaeger, S. Peacock, L. C. Mayorga, K. A. Bennett, M. López-Morales, E. M. May, Z. Rustamkulov, J. A. Valenti, J. I. Adams Redai, M. K. Alam, N. E. Batalha, G. Fu, J. Gonzalez-Quiles, A. N. Highland, E. Kruse, J. D. Lothringer, K. N. Ortiz Ceballos, K. S. Sotzen, H. R. Wakeford, High tide or riptide on the cosmic shoreline? A water-rich atmosphere or stellar contamination for the warm super-earth GJ 486b from JWST observations. *Astrophys. J. Lett.* **948**, L11 (2023).

44. E. M. May, R. J. MacDonald, K. A. Bennett, S. E. Moran, H. R. Wakeford, S. Peacock, J. Lustig-Yaeger, A. N. Highland, K. B. Stevenson, D. K. Sing, L. C. Mayorga, N. E. Batalha, J. Kirk, M. López-Morales, J. A. Valenti, M. K. Alam, L. Alderson, G. Fu, J. Gonzalez-Quiles, J. D. Lothringer, Z. Rustamkulov, K. S. Sotzen, Double trouble: Two transits of the super-earth GJ 1132 b observed with JWST NIRSpec G395H. *Astrophys. J. Lett.* **959**, L9 (2023).

45. J. Kirk, K. B. Stevenson, G. Fu, J. Lustig-Yaeger, S. E. Moran, S. Peacock, M. K. Alam, N. E. Batalha, K. A. Bennett, J. Gonzalez-Quiles, M. López-Morales, J. D. Lothringer, R. J. MacDonald, E. M. May, L. C. Mayorga, Z. Rustamkulov, D. K. Sing, K. S. Sotzen, J. A. Valenti, H. R. Wakeford, JWST/NIRCam transmission spectroscopy of the nearby sub-earth GJ 341b. *Astron. J.* **167**, 90 (2024).

46. L. Alderson, H. R. Wakeford, M. K. Alam, N. E. Batalha, J. D. Lothringer, J. Adams Redai, S. Barat, J. Brande, M. Damiano, T. Daylan, N. Espinoza, L. Flagg, J. M. Goyal, D. Grant, R. Hu, J. Inglis, E. K. H. Lee, T. Mikal-Evans, L. Ramos-Rosado, P.-A. Roy, N. L. Wallack, N. M. Batalha, J. L. Bean, B. Benneke, Z. K. Berta-Thompson, A. L. Carter, Q. Changeat, K. D. Colón, I. J. M. Crossfield, J.-M. Désert, D. Foreman-Mackey, N. P. Gibson, L. Kreidberg, M. R. Line, M. López-Morales, K. Molaverdikhani, S. E. Moran, G. Morello, J. I. Moses, S. Mukherjee, E. Schlawin, D. K. Sing, K. B. Stevenson, J. Taylor, K. Aggarwal, E.-M. Ahrer, N. H. Allen, J. K. Barstow, T. J. Bell, J. Blecic, S. L. Casewell, K. L. Chubb, N. Crouzet, P. E. Cubillos, L. Decin, A. D. Feinstein, J. J. Fortney, J. Harrington, K. Heng, N. Iro, E. M.-R. Kempton, J. Kirk, H. A. Knutson, J. Krick, J. Leconte, M. Lendl, R. J. MacDonald, L. Mancini, M. Mansfield, E. M. May, N. J. Mayne, Y. Miguel, N. K. Nikolov, K. Ohno, E. Palle, V. Parmentier, D. J. M. Petit dit de la Roche, C. Piaulet, D. Powell, B. V. Rackham, S. Redfield, L. K. Rogers, Z. Rustamkulov, X. Tan, P. Tremblin, S.-M. Tsai, J. D. Turner, M. de Val-Borro, O. Venot, L. Welbanks, P. J.

Wheatley, X. Zhang, Early release science of the exoplanet WASP-39b with JWST NIRSpec G395H. *Nature* **614**, 664–669 (2023).

47. S.-M. Tsai, E. K. H. Lee, D. Powell, P. Gao, X. Zhang, J. Moses, E. Hébrard, O. Venot, V. Parmentier, S. Jordan, R. Hu, M. K. Alam, L. Alderson, N. M. Batalha, J. L. Bean, B. Benneke, C. J. Bierson, R. P. Brady, L. Carone, A. L. Carter, K. L. Chubb, J. Inglis, J. Leconte, M. Line, M. López-Morales, Y. Miguel, K. Molaverdikhani, Z. Rustamkulov, D. K. Sing, K. B. Stevenson, H. R. Wakeford, J. Yang, K. Aggarwal, R. Baeyens, S. Barat, M. de Val-Borro, T. Daylan, J. J. Fortney, K. France, J. M. Goyal, D. Grant, J. Kirk, L. Kreidberg, A. Louca, S. E. Moran, S. Mukherjee, E. Nasedkin, K. Ohno, B. V. Rackham, S. Redfield, J. Taylor, P. Tremblin, C. Visscher, N. L. Wallack, L. Welbanks, A. Youngblood, E.-M. Ahrer, N. E. Batalha, P. Behr, Z. K. Berta-Thompson, J. Blecic, S. L. Casewell, I. J. M. Crossfield, N. Crouzet, P. E. Cubillos, L. Decin, J.-M. Désert, A. D. Feinstein, N. P. Gibson, J. Harrington, K. Heng, T. Henning, E. M.-R. Kempton, J. Krick, P.-O. Lagage, M. Lendl, J. D. Lothringer, M. Mansfield, N. J. Mayne, T. Mikal-Evans, E. Palle, E. Schlawin, O. Shorttle, P. J. Wheatley, S. N. Yurchenko, Photochemically produced SO_2 in the atmosphere of WASP-39b. *Nature* **617**, 483–487 (2023).

48. S. Constantinou, N. Madhusudhan, S. Gandhi, Early insights for atmospheric retrievals of exoplanets using JWST transit spectroscopy. *Astrophys. J. Lett.* **943**, L10 (2023).

49. D. Powell, A. D. Feinstein, E. K. H. Lee, M. Zhang, S.-M. Tsai, J. Taylor, J. Kirk, T. Bell, J. K. Barstow, P. Gao, J. L. Bean, J. Blecic, K. L. Chubb, I. J. M. Crossfield, S. Jordan, D. Kitzmann, S. E. Moran, G. Morello, J. I. Moses, L. Welbanks, J. Yang, X. Zhang, E.-M. Ahrer, A. Bello-Arufe, J. Brande, S. L. Casewell, N. Crouzet, P. E. Cubillos, B.-O. Demory, A. Dyrek, L. Flagg, R. Hu, J. Inglis, K. D. Jones, L. Kreidberg, M. López-Morales, P.-O. Lagage, E. A. Meier Valdés, Y. Miguel, V. Parmentier, A. A. A. Piette, B. V. Rackham, M. Radica, S. Redfield, K. B. Stevenson, H. R. Wakeford, K. Aggarwal, M. K. Alam, N. M. Batalha, N. E. Batalha, B. Benneke, Z. K. Berta-Thompson, R. P. Brady, C. Caceres, A. L. Carter, J.-M. Désert, J. Harrington, N. Iro, M. R. Line, J. D. Lothringer, R. J. MacDonald, L. Mancini, K. Molaverdikhani, S. Mukherjee, M. C. Nixon, A. V. Oza, E. Palle, Z. Rustamkulov, D. K. Sing,

M. E. Steinrueck, O. Venot, P. J. Wheatley, S. N. Yurchenko, Sulfur dioxide in the mid-infrared transmission spectrum of WASP-39b. *Nature* **626**, 979–983 (2024).

50. M. Mansfield, E. S. Kite, R. Hu, D. D. B. Koll, M. Malik, J. L. Bean, E. M.-R. Kempton, Identifying atmospheres on rocky exoplanets through inferred high albedo. *Astrophys. J.* **886**, 141 (2019).

51. R. Wordsworth, How likely are snowball episodes near the inner edge of the habitable zone? *Astrophys. J. Lett.* **912**, L14 (2021).

52. S. P. Quanz, M. Ottiger, E. Fontanet, J. Kammerer, F. Menti, F. Dannert, A. Gheorghe, O. Absil, V. S. Airapetian, E. Alei, R. Allart, D. Angerhausen, S. Blumenthal, L. A. Buchhave, J. Cabrera, Ó. Carrión-González, G. Chauvin, W. C. Danchi, C. Dandumont, D. Defrére, C. Dorn, D. Ehrenreich, S. Ertel, M. Fridlund, A. García Muñoz, C. Gascón, J. H. Girard, A. Glauser, J. L. Grenfell, G. Guidi, J. Hagelberg, R. Helled, M. J. Ireland, M. Janson, R. K. Kopparapu, J. Korth, T. Kozakis, S. Kraus, A. Léger, L. Leedjärv, T. Lichtenberg, J. Lillo-Box, H. Linz, R. Liseau, J. Loicq, V. Mahendra, F. Malbet, J. Mathew, B. Mennesson, M. R. Meyer, L. Mishra, K. Molaverdikhani, L. Noack, A. V. Oza, E. Pallé, H. Parviainen, A. Quirrenbach, H. Rauer, I. Ribas, M. Rice, A. Romagnolo, S. Rugheimer, E. W. Schwieterman, E. Serabyn, S. Sharma, K. G. Stassun, J. Szulágyi, H. S. Wang, F. Wunderlich, M. C. Wyatt, LIFE Collaboration, Large Interferometer For Exoplanets (LIFE). I. Improved exoplanet detection yield estimates for a large mid-infrared space-interferometer mission. *Astron. Astrophys.* **664**, A21 (2022).

53. B. S. Gaudi, S. Seager, B. Mennesson, A. Kiessling, K. Warfield, K. Cahoy, J. T. Clarke, S. Domagal-Goldman, L. Feinberg, O. Guyon, J. Kasdin, D. Mawet, P. Plavchan, T. Robinson, L. Rogers, P. Scowen, R. Somerville, K. Stapelfeldt, C. Stark, D. Stern, M. Turnbull, R. Amini, G. Kuan, S. Martin, R. Morgan, D. Redding, H. P. Stahl, R. Webb, O. Alvarez-Salazar, W. L. Arnold, M. Arya, B. Balasubramanian, M. Baysinger, R. Bell, C. Below, J. Benson, L. Blais, J. Booth, R. Bourgeois, C. Bradford, A. Brewer, T. Brooks, E. Cady, M. Caldwell, R. Calvet, S. Carr, D. Chan, V. Cormarkovic, K. Coste, C. Cox, R. Danner, J. Davis, L. Dewell, L. Dorsett, D. Dunn, M. East, M. Effinger, R. Eng, G. Freebury, J. Garcia, J. Gaskin, S. Greene, J. Hennessy, E. Hilgemann, B. Hood, W. Holota, S. Howe, P. Huang, T. Hull, R. Hunt, K. Hurd, S. Johnson,

A. Kissil, B. Knight, D. Kolenz, O. Kraus, J. Krist, M. Li, D. Lisman, M. Mandic, J. Mann, L. Marchen, C. Marrese-Reading, J. McCready, J. McGown, J. Missun, A. Miyaguchi, B. Moore, B. Nemati, S. Nikzad, J. Nissen, M. Novicki, T. Perrine, C. Pineda, O. Polanco, D. Putnam, A. Qureshi, M. Richards, A. J. Eldorado Riggs, M. Rodgers, M. Rud, N. Saini, D. Scalisi, D. Scharf, K. Schulz, G. Serabyn, N. Sigrist, G. Sikkia, A. Singleton, S. Shaklan, S. Smith, B. Southerd, M. Stahl, J. Steeves, B. Sturges, C. Sullivan, H. Tang, N. Taras, J. Tesch, M. Therrell, H. Tseng, M. Valente, D. Van Buren, J. Villalvazo, S. Warwick, D. Webb, T. Westerhoff, R. Wofford, G. Wu, J. Woo, M. Wood, J. Ziemer, G. Arney, J. Anderson, J. Maíz-Apellániz, J. Bartlett, R. Belikov, E. Bendek, B. Cenko, E. Douglas, S. Dulz, C. Evans, V. Faramaz, Y. K. Feng, H. Ferguson, K. Follette, S. Ford, M. García, M. Geha, D. Gelino, Y. Götberg, S. Hildebrandt, R. Hu, K. Jahnke, G. Kennedy, L. Kreidberg, A. Isella, E. Lopez, F. Marchis, L. Macri, M. Marley, W. Matzko, J. Mazoyer, S. McCandliss, T. Meshkat, C. Mordasini, P. Morris, E. Nielsen, P. Newman, E. Petigura, M. Postman, A. Reines, A. Roberge, I. Roederer, G. Ruane, E. Schwieterman, D. Sirbu, C. Spalding, H. Teplitz, J. Tumlinson, N. Turner, J. Werk, A. Wofford, M. Wyatt, A. Young, R. Zellem, The Habitable Exoplanet Observatory (HabEx) mission concept study final report. arXiv:2001.06683 [astro-ph.IM] (2020).

54. M. Gillon, E. Jehin, S. M. Lederer, L. Delrez, J. de Wit, A. Burdanov, V. Van Grootel, A. J. Burgasser, A. H. M. J. Triaud, C. Opitom, B.-O. Demory, D. K. Sahu, D. Bardalez Gagliuffi, P. Magain, D. Queloz, Temperate earth-sized planets transiting a nearby ultracool dwarf star. *Nature* **533**, 221–224 (2016).

55. A. P. Lincowski, V. S. Meadows, S. Zieba, L. Kreidberg, C. Morley, M. Gillon, F. Selsis, E. Agol, E. Bolmont, E. Ducrot, R. Hu, D. D. B. Koll, X. Lyu, A. Mandell, G. Suissa, P. Tamburo, Potential atmospheric compositions of TRAPPIST-1 c Constrained by JWST/MIRI Observations at 15 µm. *Astrophys. J. Lett.* **955**, L7 (2023).

56. T. P. Greene, T. J. Bell, E. Ducrot, A. Dyrek, P.-O. Lagage, J. J. Fortney, Thermal emission from the Earth-sized exoplanet TRAPPIST-1 b using JWST. *Nature* **618**, 39–42 (2023).

57. B. Benneke, T. Mikal-Evans, L. Acuna, R. Allart, T. G. Beatty, C. Cadieux, R. Cloutier, L.-P. Coulombe, R. Doyon, J. Fortney, H. A. Knutson, T. Komacek, J. Krissansen-Totton, D.

Lafreniere, R. MacDonald, Y. Miguel, P. Molliere, C. Piaulet, R. Pierrehumbert, M. Radica, P.-A. Roy, H. E. Schlichting, L. Welbanks, Exploring the existence and diversity of volatile-rich water worlds, JWST Proposal. *Cycle* **2**, 4098 (2023).

58. S. Peacock, T. Barman, E. L. Shkolnik, P. H. Hauschildt, E. Baron, Predicting the extreme ultraviolet radiation environment of exoplanets around low-mass stars: The TRAPPIST-1 system. *Astrophys. J.* **871**, 235 (2019).

59. D. J. Wilson, K. France, C. S. Froning, P. J. Wheatley, A. Youngblood, Ultraviolet photometry of TRAPPIST-1 during the next JWST observing window, HST proposal. *Cycle* **30**, 17282 (2022).

60. H. Diamond-Lowe, J. M. Mendonca, C. J. Akin, N. Allen, M. Baungaard, N. Borsato, L. A. Buchhave, A. J. Burgasser, B.-O. Demory, N. Espinoza, C. Fisher, M. Fortune, N. Gibson, A. Gressier, A. Guzman Mesa, K. Heng, J. Hoeijmakers, M. Hooton, K. Jones, D. Kitzmann, A. Lueber, E. A. Meier Valdes, B. Prinoth, A. Rathcke, M. Tian, The hot rocks survey: Testing 9 irradiated terrestrial exoplanets for atmospheres, JWST proposal. *Cycle* **2**, 3730 (2023).

61. J. E. Owen, Atmospheric escape and the evolution of close-in exoplanets. *Annu. Rev. Earth Planet. Sci.* **47**, 67–90 (2019).

62. P. B. Rimmer, C. Helling, A chemical kinetics network for lightning and life in planetary atmospheres. *Astrophys. J.* **224**, 9 (2016).

63. P. B. Rimmer, S. Rugheimer, Hydrogen cyanide in nitrogen-rich atmospheres of rocky exoplanets. *Icarus* **329**, 124–131 (2019).

64. P. B. Rimmer, S. Jordan, T. Constantinou, P. Woitke, O. Shorttle, R. Hobbs, A. Paschodimas, Hydroxide salts in the clouds of venus: Their effect on the sulfur cycle and cloud droplet pH. *Planet. Sci. J.* **2**, 133 (2021).

65. O. Shorttle, S. Jordan, H. Nicholls, T. Lichtenberg, D. J. Bower, Distinguishing oceans of water from magma on mini-neptune K2-18b. *Astrophys. J. Lett.* **962**, L8 (2024).

66. S. Jordan, O. Shorttle, P. B. Rimmer, Proposed energy-metabolisms cannot explain the atmospheric chemistry of Venus. *Nat. Commun.* **13**, 3274 (2022).

67. V. A. Krasnopolsky, Chemical kinetic model for the lower atmosphere of Venus. *Icarus* **191**, 25–37 (2007).

68. V. A. Krasnopolsky, A photochemical model for the Venus atmosphere at 47-112 km. *Icarus* **218**, 230–246 (2012).

69. W. F. Huebner, J. Mukherjee, Photoionization and photodissociation rates in solar and blackbody radiation fields. *Planet. Space Sci.* **106**, 11–45 (2015).

70. H. Keller-Rudek, G. K. Moortgat, R. Sander, R. Sörensen, The MPI-Mainz UV/VIS spectral atlas of gaseous molecules of atmospheric interest. *Earth Syst. Sci. Data* **5**, 365–373 (2013).

71. R. Wordsworth, A. H. Knoll, J. Hurowitz, M. Baum, B. L. Ehlmann, J. W. Head, K. Steakley, A coupled model of episodic warming, oxidation and geochemical transitions on early Mars. *Nat. Geosci.* **14**, 127–132 (2021).

72. I. E. Gordon, L. S. Rothman, R. J. Hargreaves, R. Hashemi, E. V. Karlovets, F. M. Skinner, E. K. Conway, C. Hill, R. V. Kochanov, Y. Tan, P. Wcisło, A. A. Finenko, K. Nelson, P. F. Bernath, M. Birk, V. Boudon, A. Campargue, K. V. Chance, A. Coustenis, B. J. Drouin, J.-M. Flaud, R. R. Gamache, J. T. Hodges, D. Jacquemart, E. J. Mlawer, A. V. Nikitin, V. I. Perevalov, M. Rotger, J. Tennyson, G. C. Toon, H. Tran, V. G. Tyuterev, E. M. Adkins, A. Baker, A. Barbe, E. Canè, A. G. Császár, A. Dudaryonok, O. Egorov, A. J. Fleisher, H. Fleurbaey, A. Foltynowicz, T. Furtenbacher, J. J. Harrison, J.-M. Hartmann, V.-M. Horneman, X. Huang, T. Karman, J. Karns, S. Kassi, I. Kleiner, V. Kofman, F. Kwabia-Tchana, N. N. Lavrentieva, T. J. Lee, D. A. Long, A. A. Lukashevskaya, O. M. Lyulin, V. Y. Makhnev, W. Matt, S. T. Massie, M. Melosso, S. N. Mikhailenko, D. Mondelain, H. S. P. Müller, O. V. Naumenko, A. Perrin, O. L. Polyansky, E. Raddaoui, P. L. Raston, Z. D. Reed, M. Rey, C. Richard, R. Tóbiás, I. Sadiek, D. W. Schwenke, E. Starikova, K. Sung, F. Tamassia, S. A. Tashkun, J. Vander Auwera, I. A. Vasilenko, A. A. Vigasin, G. L. Villanueva, B. Vispoel, G. Wagner, A. Yachmenev, S. N.

Yurchenko, The HITRAN2020 molecular spectroscopic database. *J. Quant. Spectrosc. Radiat. Transf.* **277**, 107949 (2022).

73. R. Wordsworth, Y. Kalugina, S. Lokshtanov, A. Vigasin, B. Ehlmann, J. Head, C. Sanders, H. Wang, Transient reducing greenhouse warming on early Mars. *Geophys. Res. Lett.* **44**, 665–671 (2017).

74. A. S. Ackerman, M. S. Marley, Precipitating condensation clouds in substellar atmospheres. *Astrophys. J.* **556**, 872–884 (2001).

75. P. Mollière, J. P. Wardenier, R. van Boekel, T. Henning, K. Molaverdikhani, I. A. G. Snellen, petitRADTRANS. A Python radiative transfer package for exoplanet characterization and retrieval. *Astron. Astrophys.* **627**, A67 (2019).

76. S. A. Clough, M. J. Iacono, J.-L. Moncet, Line-by-line calculations of atmospheric fluxes and cooling rates: application to water vapor. *J. Geophys. Res.* **97**, 15761–15785 (1992).

77. J. M. Mendonça, P. L. Read, C. F. Wilson, C. Lee, A new, fast and flexible radiative transfer method for Venus general circulation models. *Planet. Space Sci.* **105**, 80–93 (2015).

78. B. P. Briegleb, Delta-eddington approximation for solar radiation in the NCAR community climate model. *J. Geophys. Res.* **97**, 7603–7612 (1992).

79. J. H. Joseph, W. J. Wiscombe, J. A. Weinman, The delta-Eddington approximation for radiative flux transfer. *J. Atmos. Sci.* **33**, 2452–2459 (1976).

80. P. Mollière, T. Stolker, S. Lacour, G. P. P. L. Otten, J. Shangguan, B. Charnay, T. Molyarova, M. Nowak, T. Henning, G.-D. Marleau, D. A. Semenov, E. van Dishoeck, F. Eisenhauer, P. Garcia, R. Garcia Lopez, J. H. Girard, A. Z. Greenbaum, S. Hinkley, P. Kervella, L. Kreidberg, A.-L. Maire, E. Nasedkin, L. Pueyo, I. A. G. Snellen, A. Vigan, J. Wang, P. T. de Zeeuw, A. Zurlo, Retrieving scattering clouds and disequilibrium chemistry in the atmosphere of HR 8799e. *Astron. Astrophys.* **640**, A131 (2020).

81. E. Alei, B. S. Konrad, D. Angerhausen, J. L. Grenfell, P. Mollière, S. P. Quanz, S. Rugheimer, F. Wunderlich, LIFE Collaboration, Large Interferometer For Exoplanets (LIFE). V. Diagnostic potential of a mid-infrared space interferometer for studying Earth analogs. *Astron. Astrophys.* **665**, A106 (2022).

82. Y. L. Yung, W. B. Demore, Photochemistry of the stratosphere of Venus: Implications for atmospheric evolution. *Icarus* **51**, 199–247 (1982).

83. P. Liggins, S. Jordan, P. B. Rimmer, O. Shorttle, Growth and evolution of secondary volcanic atmospheres: 2. The importance of kinetics. *J. Geophys. Res. Planets* **128**, e2022JE007528 (2023).

84. C. M. Guimond, O. Shorttle, S. Jordan, J. F. Rudge, A mineralogical reason why all exoplanets cannot be equally oxidizing. *Mon. Not. R. Astron. Soc.* **525**, 3703–3717 (2023).

85. C. J. Bierson, X. Zhang, Chemical cycling in the Venusian atmosphere: A full photochemical model from the surface to 110 km. *J. Geophys. Res. Planets* **125**, e06159 (2020).

86. W. Bains, J. J. Petkowski, P. B. Rimmer, S. Seager, Production of ammonia makes Venusian clouds habitable and explains observed cloud-level chemical anomalies. *Proc. Natl. Acad. Sci. U.S.A.* **118**, e2110889118 (2021).

87. J. B. Garvin, S. A. Getty, G. N. Arney, N. M. Johnson, E. Kohler, K. O. Schwer, M. Sekerak, A. Bartels, R. S. Saylor, V. E. Elliott, C. S. Goodloe, M. B. Garrison, V. Cottini, N. Izenberg, R. Lorenz, C. A. Malespin, M. Ravine, C. R. Webster, D. H. Atkinson, S. Aslam, S. Atreya, B. J. Bos, W. B. Brinckerhoff, B. Campbell, D. Crisp, J. R. Filiberto, F. Forget, M. Gilmore, N. Gorius, D. Grinspoon, A. E. Hofmann, S. R. Kane, W. Kiefer, S. Lebonnois, P. R. Mahaffy, A. Pavlov, M. Trainer, K. J. Zahnle, M. Zolotov, Revealing the mysteries of Venus: The DAVINCI mission. *Planet. Sci. J.* **3**, 117 (2022).

88. D. Schulze-Makuch, D. H. Grinspoon, O. Abbas, L. N. Irwin, M. A. Bullock, A sulfur-based survival strategy for putative phototrophic life in the Venusian atmosphere. *Astrobiology* **4**, 11–18 (2004).

89. D. Schulze-Makuch, L. N. Irwin, The prospect of alien life in exotic forms on other worlds. *Naturwissenschaften* **93**, 155–172 (2006).

90. S. Seager, J. J. Petkowski, C. E. Carr, D. H. Grinspoon, B. L. Ehlmann, S. J. Saikia, R. Agrawal, W. P. Buchanan, M. U. Weber, R. French, P. Klupar, S. P. Worden, D. Baumgardner, Venus Life Finder Mission Team, Venus life finder missions motivation and summary. *Aerospace* **9**, 385 (2022).

91. R. O. P. Loyd, K. France, A. Youngblood, C. Schneider, A. Brown, R. Hu, A. Segura, J. Linsky, S. Redfield, F. Tian, S. Rugheimer, Y. Miguel, C. S. Froning, The MUSCLES treasury survey. V. FUV flares on active and inactive M Dwarfs. *Astrophys. J.* **867**, 71 (2018).
